# Prevalence of Trachoma in Four Evaluation Units in Yemen after Implementation of Trachoma Elimination Measures

**DOI:** 10.1080/09286586.2023.2180805

**Published:** 2023-03-13

**Authors:** Tawfik Al-Khateeb, Sami Al-Haidari, Robert Butcher, Anusha Rajamani, Mohammed Abdu Khalid Mahdy, Cristina Jimenez, Michael Dejene, Sarah Boyd, Ana Bakhtiari, Anthony W. Solomon, Adnan Thabit, Emma M. Harding-Esch, Rasheed Al-Shami

**Affiliations:** a Prevention of Blindness Program, Ministry of Public Health & Population, Sana’a, Yemen; b Neglected Tropical Diseases Directorate Ministry of Public Health & Population, Sana’a, Yemen; c Clinical Research Department, London School of Hygiene & Tropical Medicine, London, UK; d Parasitology Department, Sana’a University, Sana’a, Yemen; e Sightsavers, Haywards Heath, UK; f Sightsavers, Addis Ababa, Ethiopia; g Task Force for Global Health, Decatur, Georgia, USA; h Department of Control of Neglected Tropical Diseases, World Health Organization, Geneva, Switzerland; i Ophthalmology Department, Sana’a University, Sana’a, Yemen; j Taskforce for Trachoma Control Program, Ministry of Public Health & Population, Sana’a, Yemen

**Keywords:** Trachoma, trichiasis, prevalence, Yemen, elimination, blindness, neglected tropical disease

## Abstract

**Background:**

In baseline trachoma prevalence surveys, six districts in two governorates of Yemen were identified as requiring interventions. We set out to estimate the prevalence of trachoma 6 −12 months after one round of antibiotic mass drug administration (MDA) and implementation of measures to encourage facial cleanliness.

**Methods:**

A population-based prevalence survey was conducted in each of the four evaluation units in October 2019. Contemporary World Health Organization recommendations for trachoma surveys were followed. Participants were selected using a two-stage cluster sampling process. The prevalence of inflammatory and late-stage trachoma was measured through eye examination. Water, sanitation, and hygiene facility access among visited households was estimated.

**Results:**

The prevalence of trachomatous inflammation—follicular (TF) in 1–9-year-olds per EU was <5.0% in three EUs (Al Mighlaf, Al Munirah, and As Salif; Az Zaydiyah; and Mudhaykhirah districts) and 5.0–9.9% in one EU (Far Al Udayn District). The prevalence of TT unknown to the health system in ≥15-year-olds per EU was <0.2% in all four EUs. Per EU, the proportion of households with an improved drinking water source ranged from 40% to 100%; access to an improved drinking water source within 30-minute return journey of the household ranged from 45% to 100%; and with an improved latrine ranged from 32% to 83%.

**Conclusion:**

An additional round of antibiotic MDA should be administered in Far Al Udayn district before it is resurveyed. In the other surveyed districts, pre-validation surveillance surveys should be conducted in 2 years’ time to determine if the TF prevalence <5% has been maintained.

## Introduction

Trachoma, caused by ocular infection with *Chlamydia trachomatis*, is the leading infectious cause of blindness globally.^1^ The infection is thought to spread through contact with eye and nose discharge from an infected person, transmitted on fingers, fomites, and eye-seeking flies.^2–4^ After years of repeated infection, the inside of the eyelid may become scarred, and this can cause distortion of the eyelid, resulting in eyelashes rubbing on the eyeball. The presence of one or more eyelashes touching the eyeball or evidence of recent eyelash epilation, is known as trachomatous trichiasis (TT).^5^ (The TT definition was amended in November 2018 to exclude trichiasis that affects only the lower eyelid.^6^) TT is very painful and, if untreated, can lead to the formation of corneal opacity and subsequently to irreversible blindness.^7^

The World Health Organization (WHO)-endorsed SAFE (Surgery, Antibiotics, Facial cleanliness, and Environmental improvement) strategy for trachoma elimination has been implemented in over 40 trachoma-endemic countries worldwide.^8,9^ Eyelid surgery corrects the misdirected eyelashes to prevent them from rubbing on the eyeball. Mass drug administration (MDA) of antibiotics is indicated in evaluation units (EUs, defined as the local administrative unit for healthcare management, generally having a population of 100,000–250,000^10^) where the trachomatous inflammation—follicular (TF) prevalence in 1–9-year-olds is ≥5%. The recommended strategy involves delivering oral azithromycin (or 1% tetracycline eye ointment if azithromycin is unavailable or contra-indicated) to all EU residents, with a minimum acceptable coverage target of 80%. Promotion of facial cleanliness and environmental improvement are intended to facilitate sustained reductions in transmission of the pathogen.^11^ The implementation of SAFE in order to eliminate trachoma as a public health problem is guided by the EU-level prevalence of trachoma assessed by population-based prevalence surveys before MDA (baseline surveys), 6–12 months after the last recommended MDA round (impact surveys) and 2 years after the last impact survey resulted in a TF prevalence <5% (surveillance survey). Whereas A, F, and E interventions are recommended when the prevalence of TF in 1–9-year-olds is ≥5%, community-level S services are needed when the prevalence of TT unknown to the health system in ≥15-year-olds is ≥0.2%.^11,12^

Trachoma is endemic in Yemen.^13–15^ Baseline mapping took place in two phases from 2013−2015 as part of the Global Trachoma Mapping Project (GTMP). The first phase of population-based surveys for trachoma was conducted in 2013 and covered four governorates, namely Hodeidah, Ibb, Mareb, and Al-Jawf. Six EUs (covering 25 districts) of the 15 surveyed had a TF prevalence ≥5%; two EUs (6 districts) required three annual MDA rounds and four EUs (19 districts) needed one MDA round.^14,15^ The highest prevalence of 12.6% was found in the EU covering As Sabrah, As Sayyani, and Dhi As Sufal districts of Ibb governorate.^14,15^ The second phase of baseline surveys took place between 2013 and 2015 and identified two further EUs (covering five districts of the Hajjah governorate) in need of MDA.^14^ In May 2018, one round of MDA was conducted in six districts in Hodeidah and Ibb governorates based on the results of the baseline surveys. More districts were eligible for MDA but for funding and security reasons, the program decided to select these six for the first phase of MDA. Hygiene kits (containing soap and a cloth) were distributed to all families with children alongside health promotional activities to help encourage facial cleanliness in the community. Health promotion activities included the distribution of brochures promoting regular, effective face washing and broadcast of health promotion messages on radio spots, from megaphones in cars and from mosques. Interventions were focused on changing behaviour: no water, sanitation, and hygiene (WASH) infrastructure improvement projects were carried out for the purposes of trachoma elimination between the baseline and impact surveys. The six districts in which elimination measures were implemented correspond to two of the eight EUs with a TF prevalence ≥5% during baseline mapping. They included all four districts from one pre-MDA EU in Hodeidah governorate and two of the three districts from another pre-MDA EU in Ibb governorate.

Here, we aimed to estimate the prevalence of TF in 1–9-year-olds and of TT in ≥15-year-olds in four EUs covering the six districts treated in May 2018, 6 months after receiving the recommended single MDA round. The purpose of estimating prevalence was to determine if the elimination thresholds had been met, or whether further trachoma elimination activities were required.

## Methods

### Study ethics and consent

These surveys were approved by the Ethical Committee of Sana’a University (November 2018). Tropical Data survey support for trachoma surveys was approved by the London School of Hygiene & Tropical Medicine Observational Ethics Committee (16105).

Communities were contacted in advance of the teams’ arrival. The teams were accompanied by a village guide familiar with the local community. The teams sought verbal consent from household heads to enroll households in the study. All participants aged ≥18 years were required to provide verbal consent before they could take part. Verbal consent from a parent or guardian was required before people aged <18 years could take part in the surveys.

Standard quality control and quality assurance mechanisms for trachoma surveys^16^ were implemented throughout.

### Survey design and participant selection

The surveys were designed to meet WHO standards for population-based prevalence surveys for trachoma.^17^ EUs for the survey were defined according to the WHO definition of a health district for trachoma elimination purposes. The three districts had an estimated population of 100,000–250,000 and were each allocated to their own EU. There were three other districts in Al Hodeidah governorate with a population <100,000 and which were merged into a single EU (Table 1).

Because the country is under conflict, there were security issues throughout these surveys. These were managed as diplomatically as possible to enable the surveys to continue. People carrying mobile phones could be arrested at the time of the surveys: for this reason, paper-based data collection forms were used rather than mobile-based forms. Survey schedulers kept security at the forefront of their planning and, as in similarly insecure environments,^18,19^ communicated closely with district-level health authorities before and during surveys to ensure teams were working in the safest possible way.

In each EU, we planned to recruit enough children to estimate a TF prevalence of 4% in 1–9-year-olds with a precision of 2% at the 95% confidence level. After inflating by a design effect of 2.63 to account for disease clustering^20^ and by a non-response rate inflation factor of 1.2 to account for enumerated people not taking part, we determined that sufficient households to yield 1,164 children should be visited in each EU. A two-stage cluster sampling approach was taken to recruit survey participants. The primary sampling unit was the village, and the secondary sampling unit was the household. We estimated there would be 2.03 children aged 1–9 years per household in these governorates and that 30 house-holds could reasonably be visited per village per day by a field team. Consequently, 20 villages per EU were needed to meet our sample size. All residents of the selected households aged 1 year and above were eligible to be enrolled in the survey.

### Training of graders and recorders

The training of trachoma graders and recorders followed the training system outlined in the 2017 Tropical Data Training Manual.^21^ The grader trainees were general medical practitioners. The recorder trainees were university or college graduates who had experience using Smartphones and other electronic devices. Where possible, those who participated in the previous baseline surveys were preferentially selected to take part in training. Both the grader and recorder trainees were selected based on their knowledge of the local culture, traditions, and language, and on their ability to build good relationships and demonstrate a respectful attitude when challenged by individual or community beliefs and values.

To be qualified to work as trachoma graders, selected grader trainees were required to attend a standardised four-day training course and score a kappa of ≥0.7 in grading a set of 50 images compared with a consensus grade of international trachoma grading experts, and again in grading 50 school children compared to a certified grader trainer. Recorder trainees were required to attend a standardised training course, which included training for the classification of water sources and sanitation facilities, and pass a competency assessment to work as recorders in the surveys.

After the standard Tropical Data training, both graders and recorders attended an additional training session on the use of paper data collection forms and transcription to the Tropical Data data collection tool.

### Household and clinical data collection

Upon arrival at the household, the recorder collected data on the household access to WASH facilities. Questions were taken from a version of the WHO/United Nations Children’s’ Fund (UNICEF) Joint Monitoring Programme (JMP) household questionnaire modified for trachoma surveys.^22,23^ Recorders visited water sources and toilet facilities to determine what type they were, whether toilets had a handwash facility (this was only assessed where a household had a latrine) and whether water and soap were available at the handwash station. Latrines flushing to open drains were considered unimproved.

Consenting participants had both eyes examined for TT, TF, trachomatous inflammation—intense (TI) according to the WHO’s simplified trachoma grading system.^5^ The grader used 2.5× binocular loupes and sun- or torch-light. Eyes with TT (using the then-current TT definition, which did not exclude lower eyelid trichiasis) were additionally examined for trachomatous scarring (TS); the eyelid (upper or lower) from which any misdirected eyelash originated was not recorded. Participants with TT were asked whether they had been offered surgery or epilation for their TT, and whether they had accepted. Participants who had not or could not recall being offered either intervention in the eye with TT were classified as having TT ‘unknown to the health system’.

Households in which children aged 1–9 years were absent at the time of the team’s first visit were re-visited once before the end of the day to maximise recruitment.

Household and individual data were collected onto paper versions of the Tropical Data data collection tool. Data were then transcribed into a smartphone-based version of the Tropical Data app at the end of each day by two separate recorders. Data were submitted to Tropical Data-administered servers as soon as possible once a data connection was available. The duplicate submissions were compared by a data manager, and discrepancies between the two entries were identified. Discrepancies were sent back to the data entry personnel, who checked the paper forms to confirm the correct response.

### Data analysis

EU prevalence was determined by taking the mean of the age-adjusted (TF) and age- and gender-adjusted (TT) village-level disease proportions, as previously described.^22^ Confidence intervals were generated by computationally resampling adjusted village-level proportions, with replacement, 10,000 times and taking the 2.5^th^ and 97.5^th^ centile of the resulting distribution.

Association between TF and individual and household variables was conducted using mixed effects binomial regression, with village of residence included as a random-effects variable in all models. We tested whether the addition of variables to the null model improved the model fit using likelihood ratio tests. Only one variable was significant in univariable testing, so no multivariable testing was conducted. The association between TT and individual and household variables was not tested because there were too few cases of TT.

## Results

### Participant characteristics

Fieldwork for all four surveys was completed in October 2019. A total of 11,682 people aged ≥1 year were enumerated across four EUs (range: 2,747–3,147 per EU). A total of 11,118 (95%) of those enumerated were examined, with the proportion examined ranging from 93 to 97% by EU (Table 1). Overall, 289 (2%) were absent at the time of the teams’ visit, 217 (2%) refused to take part and 58 (<1%) had another, unspecified reason for not taking part. Notably, 212/217 (98%) of those who refused to take part in the study were female. Of the people examined, 3,811 (34%) were aged 1−9 years and 5,586 (50%) were aged ≥15 years.

### Signs of trachoma

**Figure 1:**
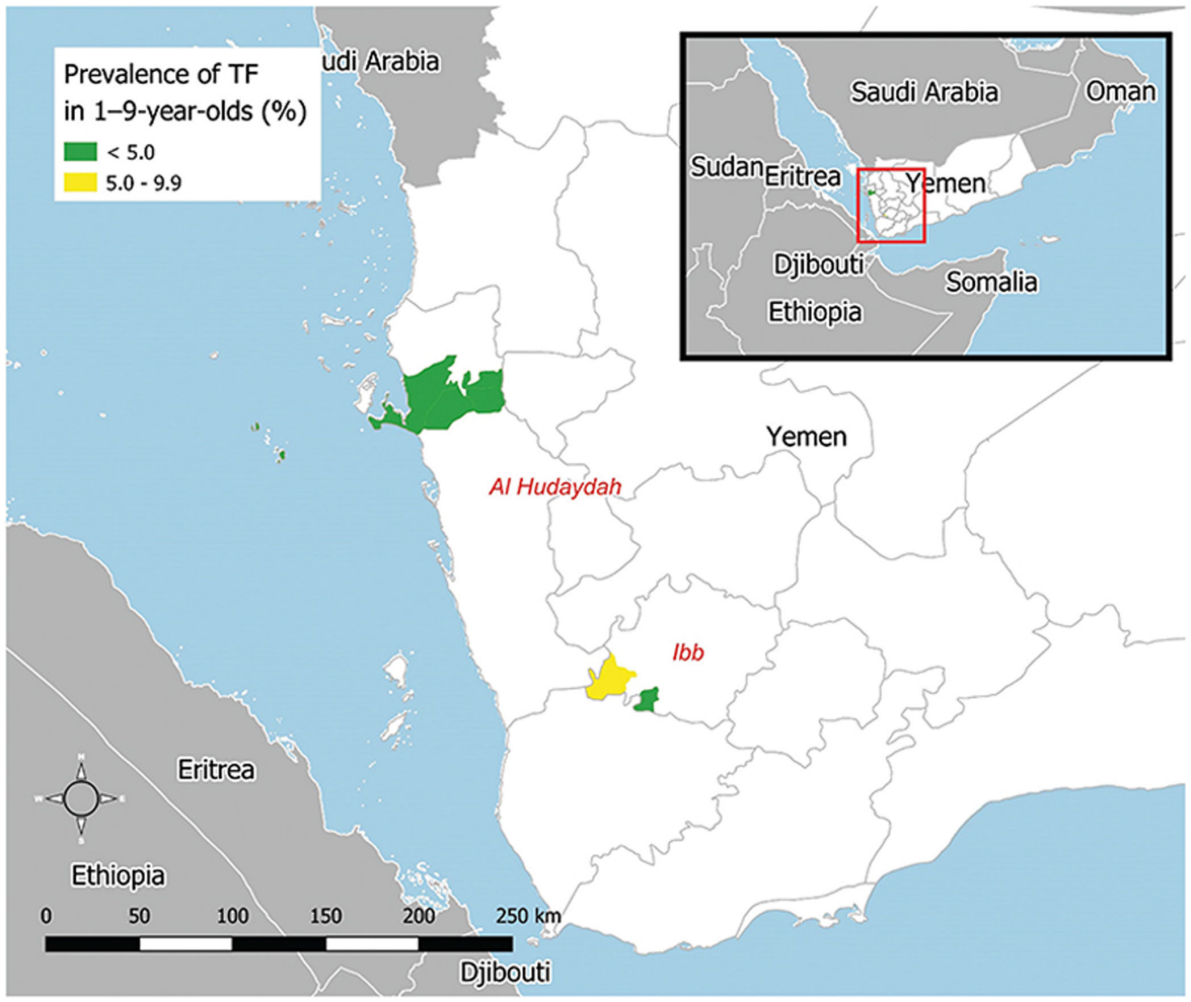
Prevalence of trachomatous inflammation—follicular (TF) in 1−9-year-olds in trachoma impact surveys of four evaluation units of Yemen, October 2019. The prevalence of trachomatous trichiasis (TT) unknown to the health system in ≥15-year-olds was <0.2% in all EUs surveyed. The boundaries and names shown and the designations used on this map do not imply the expression of any opinion whatsoever on the part of the authors, or the institutions with which they are affiliated, concerning the legal status of any country, territory, city or area or of its authorities, or concerning the delimitation of its frontiers or boundaries.

**Figure 2:**
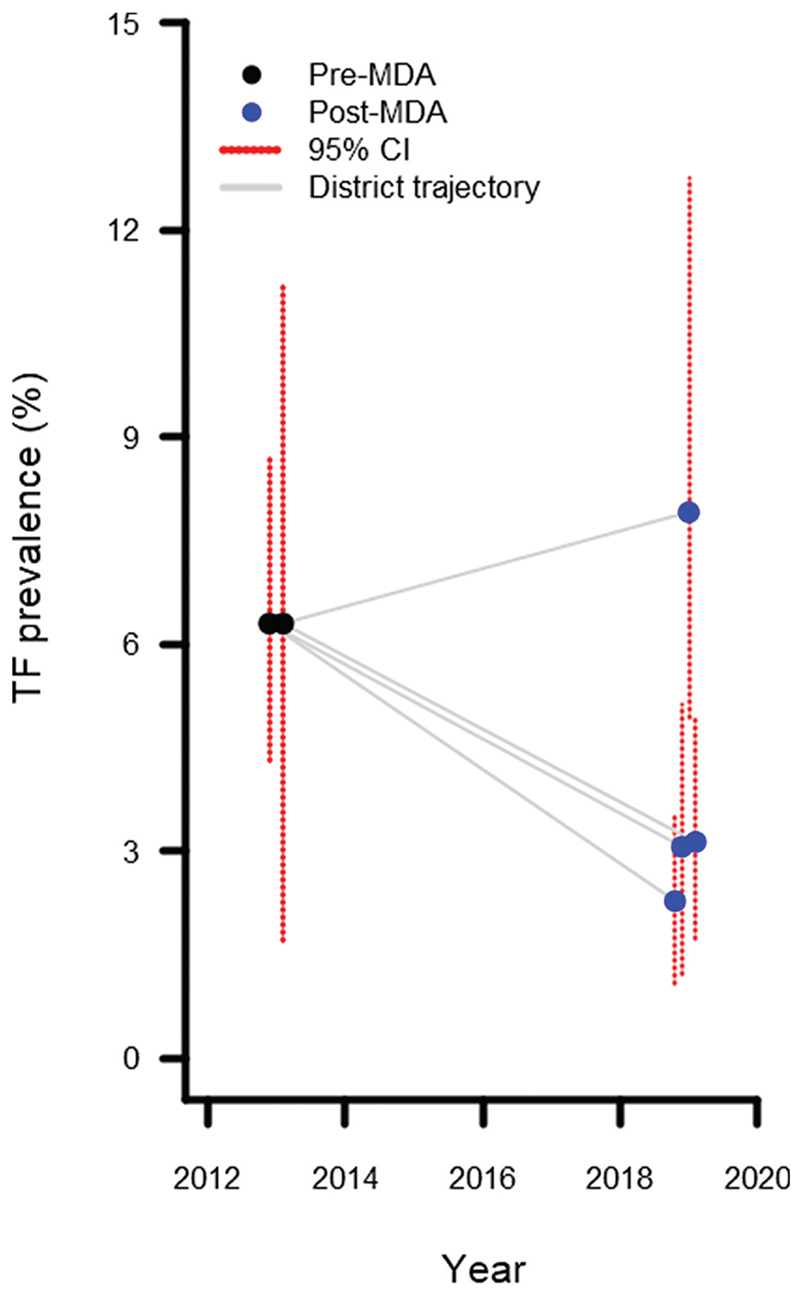
Change in prevalence of trachomatous inflammation—follicular (TF) in 1−9-year-olds following one round of antibiotic mass drug administration. X-axis coordinates have been artificially adjusted to prevent overlap and allow confidence intervals to be seen more clearly. Pre-MDA prevalence estimates are taken from Thabit *et al*., 2018^14^. Pre-MDA prevalence estimates in Al Hodeidah governate are from four districts surveyed as a single EU: Al Mighlaf, Al Munirah, As Salif and Az Zaydiyah. Prevalence estimates from Ibb governate are from three districts surveyed as a single EU, one of which was not re-surveyed in this series: Al Udayn (not resurveyed), Far Al Udayn and Mudhaykhirah. Because the pre-MDA and post-MDA EU boundaries are not identical, potential changes in prevalence between time points are not interpretable.

A total of 358 individuals were identified with TF in at least one eye, of whom 171 were aged 1−9 years (Table 2). There were 16 people with TI in at least one eye, of whom 6 were aged 1−9 years. Just over half (94/171) of TF cases in 1−9-year-olds were bilateral. The EU-level prevalence of TF in 1−9-year-olds ranged from 2.3 to 7.9%. The TF prevalence was <5% in three of the four surveyed EUs and 5.0−9.9% in one EU (Table 2, Figure 1). Three post-MDA EUs had a lower point prevalence of TF in 1−9-year-olds than that of the EU they were surveyed as previously; one had a higher prevalence (Figure 2).

There were nine people with TT identified among 5,586 people aged ≥15 years examined (Table 3). Four of those individuals with TT were women. Three of the nine individuals were affected in both eyes. Of the nine people with TT, seven also had TS and eight reported not being offered or not remembering being offered epilation or surgery. The prevalence of TT unknown to the health system was <0.2% in all EUs surveyed (Table 3).

### Water, sanitation, and hygiene access

A total of 2,417 households were visited across the four EUs. Overall, 1,884 (78%) had an improved drinking water source, 1,851 (77%) had a drinking water source within a 30-minute return journey of the house, and 1,321 (55%) had an improved latrine. The EU-level findings are presented in Table 4. The coverage of improved water sources and water sources <30 minutes from the household was noticeably lower in Far Al Udayn compared with the other EUs. Overall, 470 (19%) households practiced open defecation. Among households with a latrine (n = 1,947), 422 (22%) had a handwash station of which 323 (77%) had water and soap available at the time of the teams’ visit. TF was more common in households with an unimproved or surface washing water source compared with an improved washing water source (odds ratio: 2.1, 95% confidence interval: 1.1−3.8; p=0.026; Supplementary Table 1).

## Discussion

Interventions to reduce the prevalence of trachoma have been implemented in a number of districts of Yemen following surveys throughout the country undertaken from 2013 to 2015.^14^ Here, we report TF prevalence in the first of these districts to be resurveyed post-MDA. Three of the four EUs surveyed here, covering five districts with a collective population estimate of almost 380,000 people, have now recorded a TF prevalence of <5%, indicating MDA can be stopped and surveillance surveys conducted after 2 further years. One EU (Far Al Udayn, with an estimated population of approximately 125,000 people) had a TF prevalence of 7.9%, suggesting one additional round of MDA is needed before a repeat impact survey is conducted.^25,26^

Figure 2, which presents a change in TF prevalence following one MDA round, may be interpreted as suggesting that TF prevalence increased following MDA in one district and decreased in the others. The evidence here is insufficient to make either claim, as EU boundaries changed between pre- and post-MDA surveys. We do not feel that to be a limitation of this paper, as the primary intention of the surveys was to help programmatic planning going forward, rather than to compare with previous trachoma estimates.

The prevalence of TT unknown to the health system in ≥15-year-olds was <0.2% in all four EUs surveyed. However, WHO recommendations on TT prevalence surveys suggest that a minimum sample of 2,818 people or 30 primary-stage clusters per EU is required to estimate TT prevalence with enough precision to claim the elimination target has been achieved.^27^ We did not meet that minimum sample size in these surveys, so must assume the prevalence estimates are imprecise. Indeed, the upper bound of the confidence intervals around our prevalence estimates in the two EUs where TT cases were identified exceeded 0.2%. Geostatistical modelling, as recently applied to TT prevalence data from Ethiopia,^28^ may allow us to derive sufficient precision from the existing data presented here. In the meantime, active TT case findings should continue in the surveyed areas. A sustainable strategy to identify and manage incident cases in the longer term should also be developed to ensure those exposed to ocular *C. trachomatis* infection during their childhood can be effectively treated. This is also one of the criteria to achieve elimination of trachoma as a public health problem.^29^

Access to WASH facilities in the areas surveyed was variable. There was some evidence of a relationship between TF and living in a household without an improved washing water source. It is also interesting to note that coverage of drinking water access was lowest in the EU with the highest TF prevalence. However, the relationship between trachoma and water use is likely to be much more complex than could be captured in a survey like this, and a linear relationship between distance to water source and trachoma prevalence is unlikely.^30–32^ Lower coverage of easy access to improved water facilities is likely to be a general indication of a comparatively less economically developed area. Trachoma elimination activities in Yemen have, to date, focused on encouraging behaviour change. These should continue to be implemented in these regions to maintain the gains made by interventions so far. Targeted WASH infrastructure improvement may enable more regular face washing and may have additional health and socioeconomic benefits. This should be considered as a component of future trachoma elimination measures.

The findings here are somewhat different from findings from other contemporary reports on access to WASH infrastructure in Yemen. For example, a report from the Yemen WASH Cluster estimated <20% of the population to have access to improved water sources in Al Hodeidah governorate,^33^ whereas we found >80% of households to have an improved water source. There is heterogeneity in the methods used to estimate WASH infrastructure coverage, which could explain this difference. Moving towards integration of data collection or at least ensuring methodological standardisation is important for monitoring progress of the E component of SAFE and is likely to have benefits for other sectors, too.^24,34,35^

These surveys were affected by security challenges, for example, there were many road checkpoints during the fieldwork. In addition, paper-based data collection had to be used rather than mobile-based forms. Both of these factors increased survey time, with budget implications. The advantages of electronic data collection have been previously outlined,^22,36^ but it was not possible to employ this methodology for this survey series due to insecurity. The use of paper forms resulted in additional personnel costs in order to conduct double data entry and resolve discrepancies, and delays from data collection to having clean data available for analysis. This limited our ability to provide data-related feedback to teams whilst they were still in the field, and meant teams were unable to proceed with the field-work schedule as they waited for data entry from the previous EU to be completed. There were other limitations to these findings: our survey sample size target was not met in two EUs (covering Al Mighlaf, Al Munirah and As Salif, and Az Zaydiyah). This would have resulted in a slightly reduced precision in the TF prevalence estimate compared to what we planned. We also did not survey enough villages per EU to make a TT estimate with the precision to declare the elimination threshold to have decisively been reached, as described above.

Looking forward, the programme plans to continue with impact surveys in the remaining EUs, which have received the requisite number of MDA rounds. This should include Al Udayn district, which was included in the GTMP Ibb governorate at baseline but not included in the impact surveys presented here. Surveillance surveys should be planned in the districts of Ibb and Al Hodeidah, which have now met the elimination targets, whilst taking into account security challenges, which will likely disrupt programme planning and implementation in some parts of the country. Shabwah and Amran governorates were too insecure for survey activities during the GTMP: baseline surveys are still needed there.

## Conclusion

The reduction of TF below the elimination target in three of four surveyed EUs is a promising step in Yemen’s progress towards elimination of trachoma as a public health problem. Future impact surveys conducted following antibiotic MDA will determine the need for additional MDA rounds. Surgical services, facial cleanliness, and environmental improvement should all continue to be implemented to ensure the sustainability of trachoma elimination.

## Supplementary Material

Supplemental data for this article can be accessed online at https://doi.org/10.1080/09286586.2023.2180805

Supplementary Table 1

## Figures and Tables

**Table 1:** Population enumerated, examined, and reasons for nonparticipation during trachoma impact surveys in two governates of Yemen, October 2019.

Governate	Districts	EU ID	Enumerated, n	Absent, n	Refused, n	Other non-specified reason for not taking part, n	Examined, n	Examined female, n (%)
Al Hodeidah	Al Mighlaf, Al Munirah and As Salif	80862	2,747	56	33	0	2,658	1,313 (49)
Al Hodeidah	Az Zaydiyah	80863	3,020	70	75	4	2,871	1,320 (46)
Ibb	Far Al lldayn	80864	2,768	90	59	49	2,570	1,217 (47)
Ibb	Mudhaykhirah	80865	3,147	73	50	5	3,019	1,602 (53)

EU ID: Evaluation unit identifier; MDA: mass drug administration; TF: trachomatous inflammation—follicular.

**Table 2:** Prevalence of trachomatous inflammation—follicular (TF) in children aged 1−9 years in trachoma impact surveys of two governates of Yemen, October 2019.

Governate	Districts	Impact survey, 2019				2013 pre-MDA prevalence of TF in 1–9-year-olds (%, 95% CI)^13^
		Examined children aged 1–9 years	Children aged 1–9 years with TF in at least one eye (%)	Children aged 1–9 years with TI in at least one eye (%)	Adjusted prevalence of TF in children aged 1–9 years* (%, 95% CI)*	
Al Hodeidah	Al Mighlaf, Al Munirah and As Salif	811	22 (3)	0 (0)	2.3 (1.1–3.5)	6.3 (4.3–8.7)**
Al Hodeidah	Az Zaydiyah	942	26 (3)	2 (<1)	3.1 (1.2–5.1)	6.3 (4.3–8.7)**
Ibb	Far Al udayn	983	81 (8)	0 (0)	7.9 (4.9–12.7)	6.3 (1.7–11.2) ^¶^
Ibb	Mudhaykhirah	1,075	42 (4)	4 (<1)	3.1 (1.8–4.9)	6.3 (1.7–11.2)^ ¶^

* Adjusted in one-year age bands according to the 2004 census.^24^

** Prevalence in four districts in Al Hodeidah governate surveyed as a single EU: Al Mighlaf, Al Munirah, As Salif and Az Zaydiyah.^13,14^

¶ Prevalence in three districts in Ibb governate surveyed as a single EU: Al Udayn, Far Al Udayn and Mudhaykhirah.^13,14^ CI: confidence interval; TI: trachomatous inflammation—intense.

**Table 3:** Prevalence of trachomatous trichiasis (TT) in people aged ≥15 years in trachoma impact surveys, two governates of Yemen, October 2019.

Governate	Districts	Impact survey, 2019					2013 pre-MDA prevalence of TT in ≥ 15-year-olds (%, 95% CI)^13^
		Examined aged ≥15 years	People aged ≥15 years with TT in at least one eye (%)	People aged ≥15 years with TT and TS in at least one eye	People aged ≥15 years with TT unknown to the health system in at least one eye	Adjusted prevalence of TT unknown to the health system in people aged ≥15 years (%, 95% CI)*	
Al Hodeidah	Al Mighlaf, Al Munirah and As Salif	1,472	5 (0.3)	4 (0.3)	5 (0.3)	0.19 (0.00–0.45)	0.1 (0.0–0.1)**
Al Hodeidah	Az Zaydiyah	1,512	0 (0.0)	0 (0.0)	0 (0.0)	0.00 (-)	0.1 (0.0–0.1)**
Ibb	Far Al Udayn	1.171	1 (0.1)	0 (0.0)	0 (0.0)	0.00 (-)	0.17 (0.0–0.5) ^¶^
Ibb	Mudhaykhirah	1,431	3 (0.2)	3 (0.2)	3 (0.2)	0.15 (0.00–0.46)	0.17 (0.0–0.5)^ ¶^

Impact survey, 2019

* Adjusted in five-year age and gender bands according to the 2004 census.^24^

** Prevalence in four districts in Al Hodeidah governate surveyed as a single EU: Al Mighlaf, Al Munirah, As Salif and Az Zaydiyah.^13,14^

¶ Prevalence in three districts in Ibb governate surveyed as a single EU: Al Udayn, Far Al Udayn and Mudhaykhirah.^13,14^ TS: trachomatous scarring.

**Table 4:** Household water, sanitation and hygiene access, trachoma impact surveys, two governates of Yemen, October 2019.

Governate	Districts	Impact survey, 2019						Pre-MDA survey, 2013			
		Villages	Households visited	Households with improved drinking water source, n (%)	Households with a drinking water source within 30-minute return journey of the household, n (%)	Households with an improved latrine, n (%)		Households visited	Households with improved drinking water source, n (%)	Households with a drinking water source within 30-minute return journey of the household, n (%)	Households with an improved latrine, n (%)
Al Hodeidah	Al Mighlaf, Al Munirah and As Salif	20	600	510 (85)	475 (79)	193 (32)		607	346 (57)	254 (42)	445 (73)
Al Hodeidah	Az Zaydiyah	20	600	600 (100)	598 (>99)	432 (72)					
Ibb	Far Al Udayn	20	600	242 (40)	267 (45)	495 (83)		827	759 (92)	627 (76)	587 (71)
Ibb	Mudhaykhirah	20	617	532 (86)	511 (83)	201 (33)					
