# Supplementary material for: Prevalence of Trachoma in Four Evaluation Units in Yemen after Implementation of Trachoma Elimination Measures
Source: Ophthalmic Epidemiol. Author manuscript; Available in PMC 2026 Mar 9. (PMC7618836; doi:10.1080/09286586.2023.2180805)
Supplement: Supplementary Table 1 [file EMS212687-supplement-Supplementary_Table_1.docx]

| **Supplementary table 1.** Univariable binomial logistic regression analysis of relationship between having trachomatous inflammation**—**follicular (TF) and individual- and household-level variables. Analyses include cluster of residence as a random-effects variable. Association tested using likelihood ratio test between models with and without fixed effect variable of interest. One child was excluded from this analysis as their household water facility could not be classified in the field. | | | | | |
| --- | --- | --- | --- | --- | --- |
|  |  |  |  |  |  |
| **Variable** | **Level** | **No TF** | **TF** | **Odds ratio (95% CI)** | **p-value** |
| Age group (years) | 1−3 | 1,022 | 41 | Reference | 0.578 |
|  | 4−6 | 1,424 | 69 | 1.2 (0.8−1.7) |  |
|  | 7−9 | 1,193 | 61 | 1.2 (0.8−1.9) |  |
| Gender | Male | 1,916 | 97 | Reference | 0.365 |
|  | Female | 1,723 | 74 | 0.9 (0.6−1.2) |  |
| Children under 10 years per household | 01-Feb | 1,795 | 86 | Reference | 0.569 |
|  | ≥3 | 1,844 | 85 | 0.9 (0.7−1.3) |  |
| Household washing water source status | Improved | 2,973 | 110 | Reference | 0.026 |
|  | Unimproved or surface | 666 | 61 | 2.1 (1.1−3.8) |  |
| Return journey to household washing water source | <30 minutes | 2,875 | 112 | Reference | 0.1 |
|  | ≥30 minutes | 764 | 59 | 1.6 (0.9−2.8) |  |
| Household latrine status | Improved | 2,555 | 127 | Reference | 0.531 |
|  | Unimproved | 397 | 23 | 0.9 (0.5−1.7) |  |
|  | Open | 687 | 21 | 0.7 (0.4−1.3) |  |
| Household latrine ownership | Private or shared | 2,956 | 150 | Reference |  |
|  | Open | 683 | 21 | 0.7 (0.4−1.3) | 0.284 |
| CI: confidence interval. | | | | | |
